# Marked reduction of SARS-CoV-2 infection and improved recovery following supplementation with a probiotic mix of four strains and two strains of *Bifidobacterium breve* in hamsters

**DOI:** 10.1128/aem.00648-25

**Published:** 2025-05-12

**Authors:** Edgar Torres-Maravilla, Marine Wasniewski, Aurélie Wauquier, Alexandre Servat, Evelyne Picard-Meyer, Elodie Monchatre-Leroy, Sandrine Auger, Sophie Holowacz, Franck Boué, Philippe Langella, Elsa Jacouton, Anne-Judith Waligora-Dupriet

**Affiliations:** 1 Université Paris-Saclay, INRAe, AgroParisTech, Micalis Institute 27048, Jouy-en-Josas, France; 2 ANSES, LRFSN, Laboratoire de la rage et de la faune sauvage de Nancy 355165 , Malzéville, France; 3 INSERM, UMRS1139, 3PHM, Université de Paris 555089, Paris, France; 4 PiLeJe, Paris, France; University of Nebraska-Lincoln, Lincoln, Nebraska, USA

**Keywords:** probiotics, SARS-CoV-2, pulmonary infection, covid, bifidobacteria, *Lactobacillus*

## Abstract

Only a few studies have shown that certain probiotics have beneficial effects on severe acute respiratory syndrome coronavirus 2 (SARS-CoV-2) infection. In this study, two strains of *Bifidobacterium breve*, CNCM I-5644 and CNCM I-5979, selected for their *in vitro* immunomodulatory properties demonstrated in a screening of 20 strains and a mixture of 4 probiotic strains selected based on its immunomodulatory and antiviral properties were evaluated in a hamster model of SARS-CoV-2 infection. Supplementation with these probiotics (7 days before plus 7 days after infection) reduced SARS-CoV-2 infection with a significantly reduced viral load in the upper respiratory tract and lungs and improved weight recovery. Probiotics also counteracted the increase in inflammatory markers and intestinal permeability. The impact of these probiotics was independent of microbiota composition and short-chain fatty acid production. Overall, these data suggest that the probiotics tested, in particular the mix containing *Bifidobacterium longum* LA101, *Lactobacillus helveticus* LA102, *Lactococcus lactis* LA103, and *Streptococcus thermophilus* LA104, can facilitate recovery from SARS-CoV-2 infection (as shown by weight regain in infected hamsters) by reducing viral load and inflammation.

**IMPORTANCE:**

Our study investigated the potential benefits of specific probiotics in fighting severe acute respiratory syndrome coronavirus 2 (SARS-CoV-2) infection (COVID-19). We tested two strains of *Bifidobacterium breve* selected based on their immune-boosting properties, along with a commercial mix of four probiotic strains chosen for its antiviral and immune-modulating effects. These probiotics were administered to hamsters over a week before and a week after infection. Supplementation with these probiotics significantly reduced the viral load in the upper respiratory tract and lungs, promoting recovery as demonstrated by the weight regain observed. In addition to reducing viral presence, the probiotics also helped lower inflammation and improved gut health by counteracting increased intestinal permeability. Our findings suggest that probiotics, particularly the mix of four strains, could support recovery from SARS-CoV-2 infection by reducing inflammation, viral load, and enhancing overall health.

## INTRODUCTION

The global pandemic of coronavirus disease-19 (COVID-19) caused by severe acute respiratory syndrome coronavirus 2 (SARS-CoV-2) has resulted in a variety of clinical manifestations from mild to severe respiratory illness in chronically ill patients.

SARS-CoV-2 infection is mediated by the attachment of spike S1 glycoprotein to angiotensin-converting enzyme 2 (ACE2) before entering the cell (1). The upper respiratory tract (URT) and lungs are the primary sites of entry and replication of SARS-CoV-2, causing symptoms of pneumonia and alveolar damage (2). Gastrointestinal disturbances have also been reported (3) and are associated with gut microbiota dysbiosis (4–6).

The rationale for the use of probiotics in COVID-19 is mainly derived from indirect evidence based on their properties of (i) stimulating the mucosal immune response, (ii) reducing intestinal permeability, and (iii) maintaining immune homeostasis (7). Only a few studies have reported the beneficial effects of probiotics on SARS-CoV-2 infection by reducing the severity and duration of symptoms, nasopharyngeal viral load, and by lowering the risk of developing respiratory failure and mortality (8–10). In addition, a number of studies have demonstrated the ability of probiotics to reduce viral infection in intestinal and respiratory cells through the antiviral interferon pathway and by decreasing inflammatory response (11–13). These studies suggest a potent probiotic prophylactic effect against SARS-CoV-2, although further research is needed to understand how probiotics specifically exert their effect.

Our objective was to explore the preventive effects of a 14-day supplementation with probiotics against SARS-CoV-2 infection in a hamster model that recapitulates the characteristics observed in mild human infection (14). Probiotics tested were two strains of *Bifidobacterium breve*, CNCM I-5644 and CNCM I-5979, selected for their properties during preliminary screening and a mixture of four strains containing *Bifidobacterium longum* LA101, *Lactobacillus helveticus* LA102, *Lactococcus lactis* LA103, and *Streptococcus thermophilus* LA104 (marketed under the names Lactibiane Référence, Lactibiane Reference, and Lactibiane Reference V by PiLeJe Laboratoire) selected for its immunomodulatory and antiviral properties (PiLeJe data on file). The mix is referred to as LR in the following paragraphs.

## RESULTS

### *In vitro* selection of bacterial strains with immunomodulatory properties

**Fig 1:**
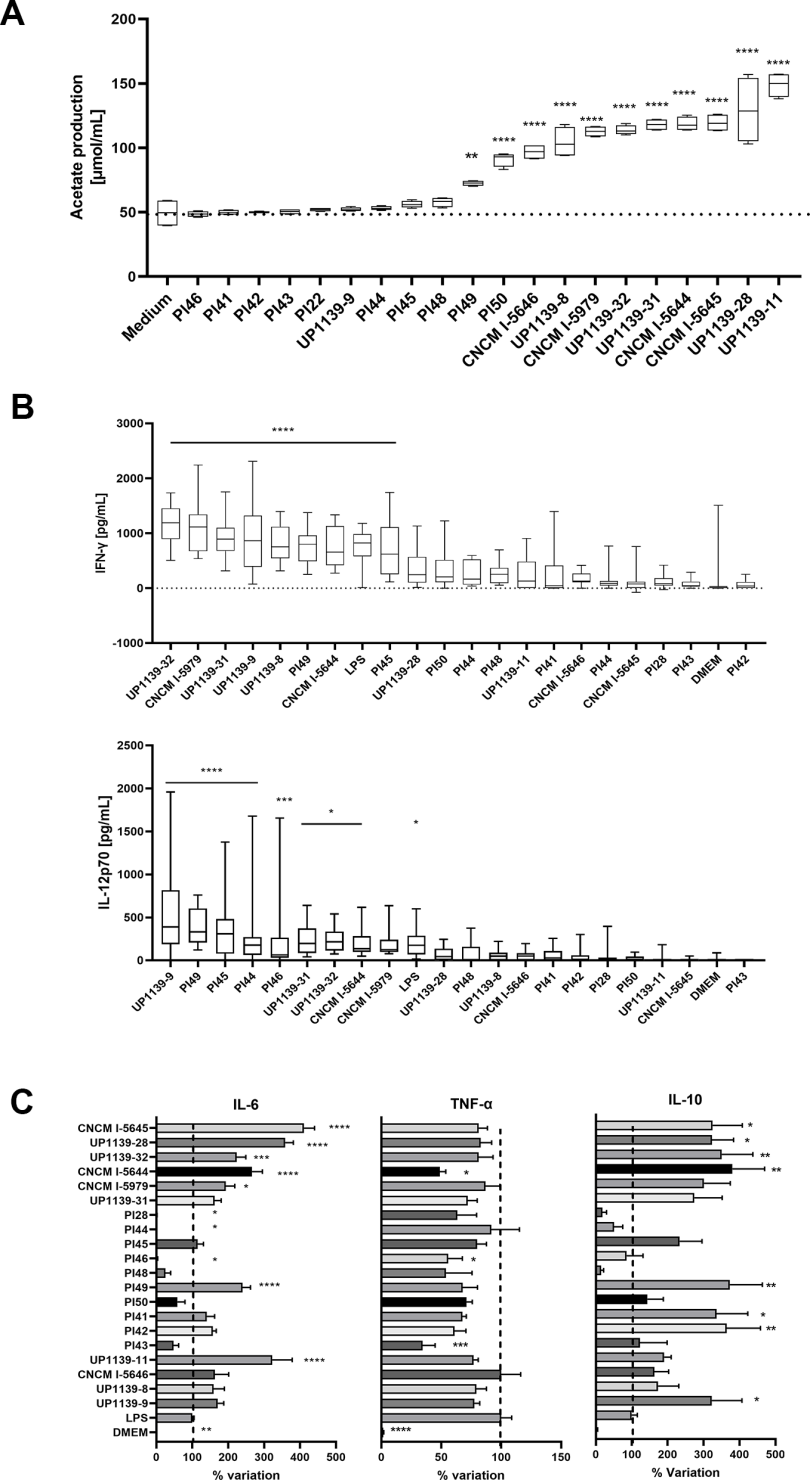
*In vitro* screening of 20 bacterial strains for immunomodulatory and antiviral effects. (**A**) Acetate production, (**B**) cytokine production by peripheral blood mononuclear cells (PBMC) after coincubation with bacteria, and (**C**) cytokine production by LPS-induced murine macrophages after coincubation with bacteria. Data were analyzed using a one-way ANOVA followed by Dunnett’s multiple comparison test, *P value < 0.05; ***P value < 0.001; ****P value < 0.0001 in comparison with negative control (Dulbecco’s modified Eagle’s medium [DMEM]).

Recent studies have highlighted the protective role of acetate in viral infection, either by improving the airway epithelial barrier (15) or enhancing the antiviral response (16). We then determined the production of acetate of 20 strains *in vitro* (Table S1). Eleven strains induced significant acetate production compared to negative control (Fig. 1A). Antiviral activity was assessed by measuring IFN-γ and IL-12p70 release from peripheral blood mononuclear cells (PBMCs). Eight strains enhanced IFN-γ production, and eight strains increased IL-12p70 (Fig. 1B). Three strains showed significant anti-inflammatory activity with a reduction of IL-6 in lipopolysaccharide (LPS)-induced murine macrophages, and three strains decreased TNF-α (Fig. 1C). Eight strains induced IL-10 production (Fig. 1C). Based on these results, strains CNCM I-5644 and CNCM I-5979 were selected for *in vivo* experiments.

### Supplementation with LR, CNCM-I 5979, or CNCM-I 5644 reduced SARS-CoV-2 infection

**Fig 2:**
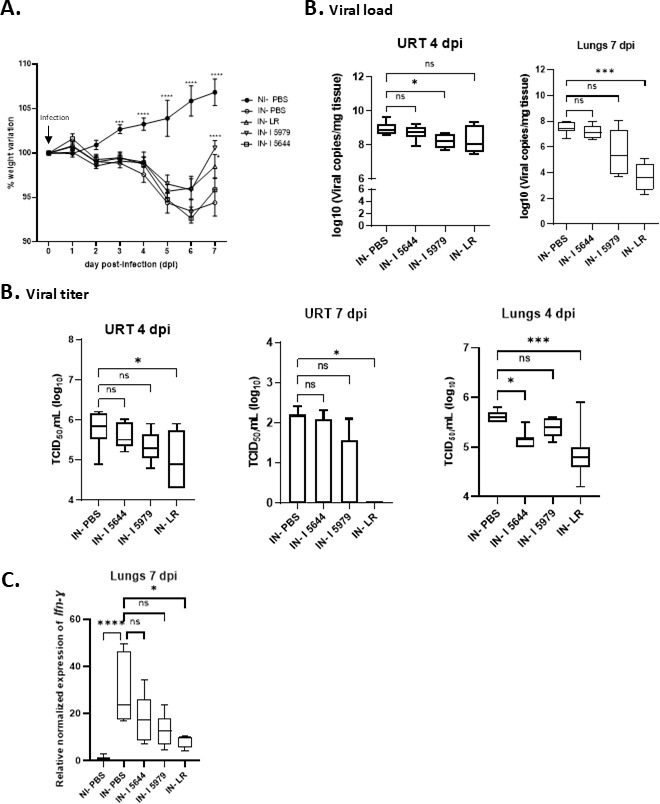
Supplementation with probiotics reduced SARS-CoV2 infection in hamsters. (**A**) Body weight variation compared to IN-PBS hamsters, (**B**) virus load and viral titer in lungs and upper respiratory tract (URT) at 4 and 7 dpi, and (**C**) *Ifn-γ* mRNA in lungs at 7 dpi. Infection was induced by intranasal inoculation of SARS-CoV-2 strain UCN1. -IN-I 5644, infected hamsters supplemented with *B. breve* CNCM I-5644; IN-I 5979, infected hamsters supplemented with *Bifidobacterium breve* CNCM I-5979; IN-LR, infected hamsters supplemented with LR (a mix of four probiotic strains); IN-PBS, infected hamsters supplemented with PBS; NI-PBS: non-infected hamsters supplemented with PBS; ns, not significant; TCID_50_, Median tissue culture infectious dose. Data were analyzed using Kruskal-Wallis test, followed by Dunn’s multiple comparisons, *P value < 0.05; ***P value < 0.001; ****P value < 0.0001.

SARS-CoV-2-infected hamsters started to significantly lose weight from 3 days post-infection (dpi) and continued to do so until 6 or 7 dpi, depending on the group, compared with non-infected hamsters (Fig. 2A). Unlike the other groups, hamsters supplemented with LR or CNCM-I 5979 began to recover their weight between 6 and 7 dpi. Supplementation with CNCM-I 5979 and CNCM-I 5644 significantly reduced the viral pulmonary load in the URT at 4 dpi and the viral titer in the lungs at 4 dpi, respectively (Fig. 2B). Supplementation with LR significantly reduced the viral load at 7 dpi in the lungs and the viral titer in the URT at 4 and 7 dpi and in the lungs at 4 dpi (Fig. 2B). SARS-CoV-2 infection caused higher *Ifn-γ* expression in the lungs at 7 dpi; only LR was able to significantly lower it (Fig. 2C).

These data confirm pulmonary SARS-CoV-2 infection and indicate that probiotic supplementation, particularly with LR, facilitates recovery—as demonstrated by weight regain—by reducing viral titer and load from 4 dpi.

### Probiotics modulated the expression of intestinal genes linked to inflammation, barrier, and antiviral response

**Fig 3:**
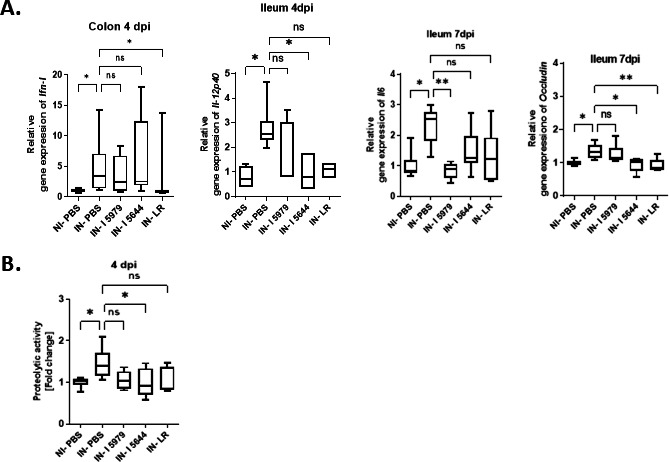
Changes in intestinal transcript levels of Ifn-I, Il-12p40, Il-6, and occludin (**A**) and fecal proteolytic activity (**B**) during SARS-CoV-2 infection. -IN-I 5644: infected hamsters supplemented with *B. breve* CNCM I-5644; IN-I 5979, infected hamsters supplemented with *Bifidobacterium breve* CNCM I-5979; IN-LR, infected hamsters supplemented with LR (a mix of four probiotic strains); IN-PBS, infected hamsters supplemented with PBS; NI-PBS, non-infected hamsters supplemented with PBS; ns, not significant. Data were analyzed using Kruskal-Wallis test, followed by Dunn’s multiple comparisons, *P value < 0.05; **P value < 0.01.

SARS-CoV-2 infection significantly increased transcripts encoding *Ifn-I* in the colon at 4 dpi, *Il-12p40* in the ileum at 4 dpi, and *Il-6* and *Occludin* in the ileum at 7 dpi (Fig. 3A). Supplementation with LR significantly reduced *Ifn-I* in the colon at 4 dpi and *Occludin* in the ileum at 7 dpi. Supplementation with CNCM I-5979 only reduced *Il-6* in the ileum at 7 dpi, and CNCM I-5644 reduced *Il-12p40* and *Occludin* in the ileum at 4 and 7 dpi, respectively. The anti-inflammatory activity of CNCM I-5644 was associated with a significant reduction in proteolytic activity, which was slightly increased at 4 dpi in infected animals compared with mock ones (Fig. 3B). The other probiotics tested tended to reduce it without reaching the significance threshold. No other regulation was observed in the intestine.

These results show that SARS-CoV-2 infection caused gut disturbances, increasing genes linked to inflammation, intestinal permeability, and antiviral response, which were partially counteracted by probiotic supplementation.

### The impact of probiotics was independent of microbiota composition and short-chain fatty acid (SCFA) production

**Fig 4:**
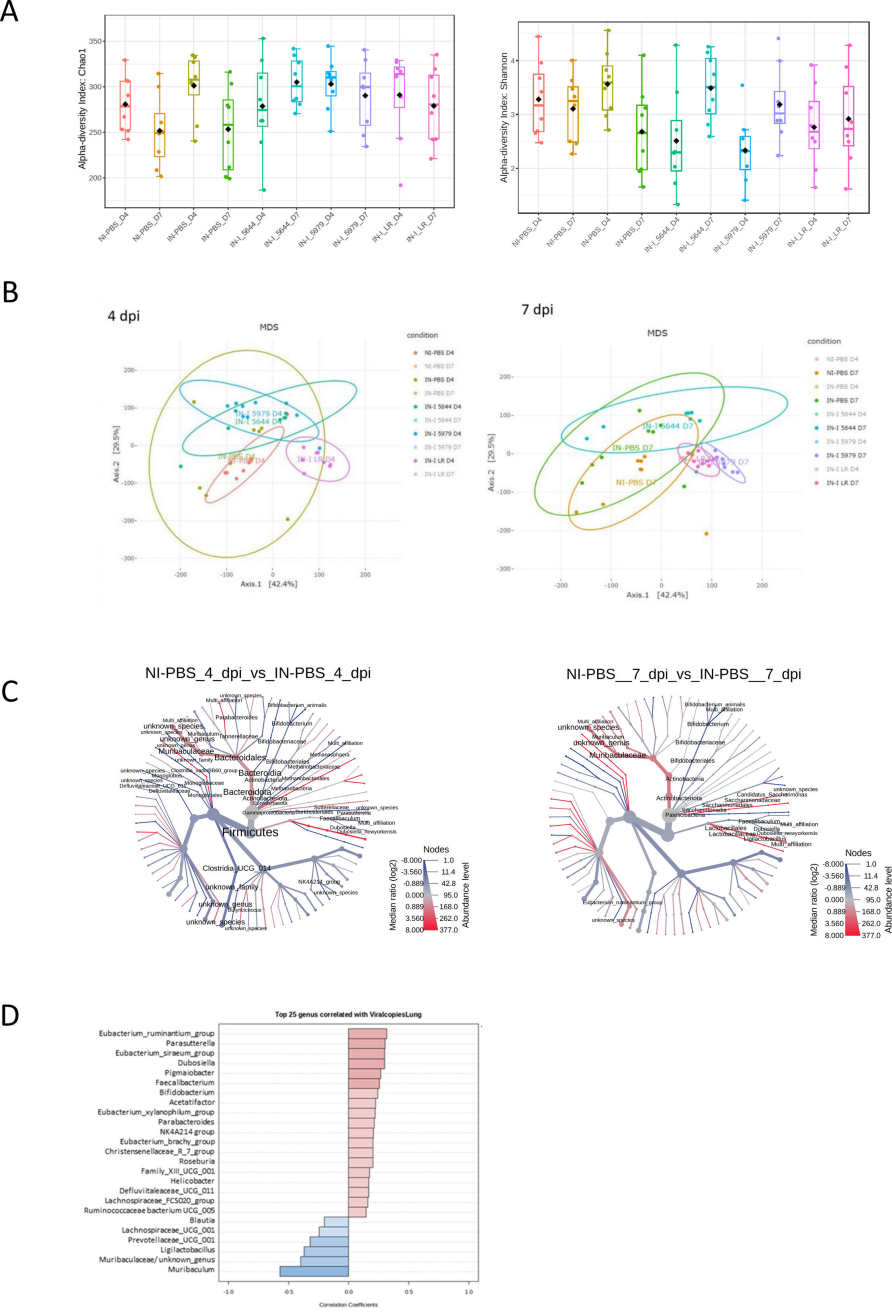
Microbiota analysis using 16S sequencing. (**A**) Alpha diversity using Chao and Shannon indexes; (**B**) beta diversity using Bray-Curtis distances; (**C**) heat tree analysis representing the taxonomic differences between the NI-PBS and IN-PBS microbial communities at 4 and 7 dpi (highlighted by the Wilcoxon rank-sum test); and (**D**) top 25 genera correlated with viral copies in the lung assessed using Spearman rank correlation. Light color non-significant, medium color significant (*P* < 0.05), dark color remains significant following FDR correction.

**Fig 5:**
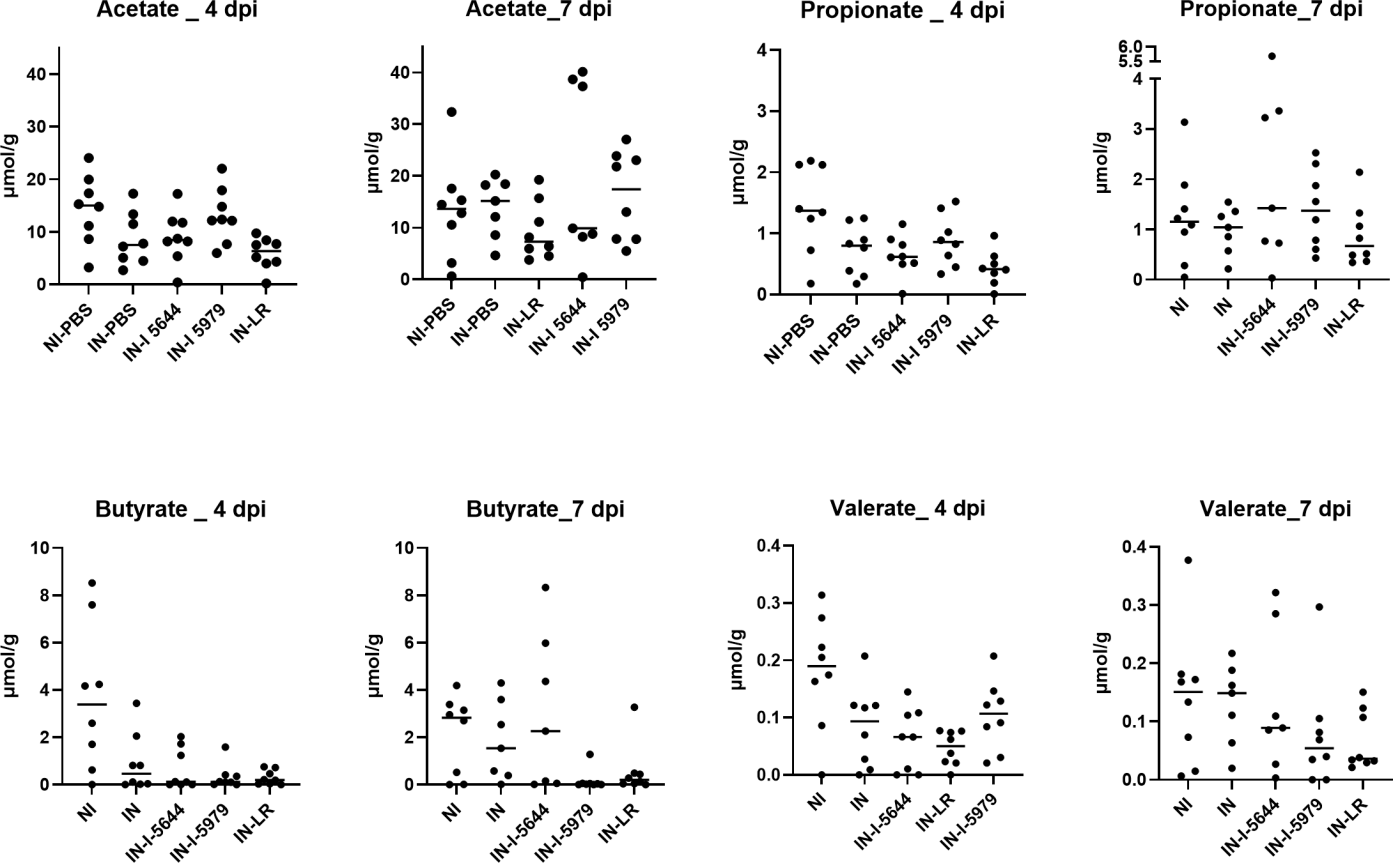
Fecal short-chain fatty acids at 4 and 7 dpi. Acetate, propionate, butyrate, and valerate were assessed by gas chromatography at 4 and 7 dpi in fecal samples.

Alpha diversity was not statistically modified during SARS-CoV-2 infection (Fig. 4A). Beta diversity plots showed clustering by treatment and time points (Fig. 4B), which led us to analyze differences in microbiota composition. At the genus level, microbiota in infected hamsters was characterized by significantly lower levels of *Ligilactobacillus* and *Muribaculum* while it contained significantly higher levels of *Dubosiella*, *Bifidobacterium*, *Parabacteroides*, *Oscillospiraceae NK4A214 group*, *Facecalibacterium,* and *Monoglobus* (Fig. 4C). There was no major modification of fecal SCFA in infected hamsters except for a trend toward a decrease at 4 dpi, which disappeared at 7 dpi (Fig. 5). Supplementation with probiotics did not significantly alter microbiota of infected hamsters nor SCFA production, which remained low overall and like that observed in infected hamsters. Spearman correlation analysis identified *Prevotellaceae*_UCG_001, *Ligilactobacillus*, unknown genus from *Muribaculaceae,* and *Muribaculum* were negatively correlated with viral copy number (*ρ* = −0.37, –0,33, −0.46, and 0.58, respectively) (Fig. 4D), the latter being also negatively correlated with lung *Ifn-γ* (*ρ* = −0.49) and ileal occludin (*ρ* = −0.35) but tended to be positively correlated with ileal *Il-6*.

## DISCUSSION

Nutraceutical compounds such as vitamins, minerals, and probiotics attracted growing interest since the beginning of the COVID-19 pandemic, particularly due to the lack of effective treatment and vaccination at that time. Five years later, evidence for the *in vivo* antiviral activity of probiotics against SARS-CoV-2 is still limited. In this study, we assessed the effects of two strains selected from *in vitro* screening and a commercial probiotic mix of four strains (LR) in a SARS-CoV-2 hamster model, taking into account the two-time windows corresponding to the early (2–4 dpi) and late (7–10 dpi) immune response observed in this model (14).

Hamsters supplemented with LR experienced faster weight recovery. In addition, LR showed the best antiviral activity with a reduction in the virus load and titer from 4 to 7 dpi in both the URT and lungs, along with a reduction of *Ifn*-γ levels in the lungs. This effect was not due to lower expression of ACE2 (tested but not shown), suggesting that the mix reduces infection via other mechanisms. Molecular docking showed the feasible bound structure between bacteriocins and RNA-dependent RNA polymerase (RdRp), the receptor-binding domain of SARS-CoV-2 (RBD) and ACE2 (17, 18). Other mechanisms, such as IgM and IgG involvement, have been described following probiotic supplementation in SARS-CoV-2-infected humans (9). Therefore, it could be interesting to address cytotoxic immunity and Ig-related immunity in order to decipher how probiotics act.

Both CNCM I-5644 and CNCM I-5979 had less effect on lung infection; however, they improved intestinal inflammation and permeability. In addition, data obtained with CNCM I-5644 confirmed the ability of the strain to reduce fecal proteolytic activity (19) that could explain the improved intestinal physiology.

Modifications of the microbiota and SCFA are coherent with alterations that were previously described, showing an alteration of the gut microbiota that correlates with disease severity (20). Interestingly, in our model, some of the known immunomodulatory potential genera were increased in infected hamsters, such as *Faecalibacterium*, *Oscillibacter*, and *Bifidobacterium.* In contrast, we observed a major decrease in *Muribaculaceae* and *Muribaculum. Muribaculaceae* is known to produce SCFA, such as butyrate and propionate, and *Muribaculum intestinalis* is able to induce adaptive immune responses during homeostasis (21). Moreover, an increased abundance of *Muribaculum* was also observed with the repair of the lung barrier in an influenza A virus infection model (22). Our probiotic strains failed to restore the gut microbiota balance and SCFA concentrations, suggesting a direct effect on the gut-lung axis without affecting the gut microbiota, which is consistent with previous work (20). However, we cannot completely rule out the absence of an effect through the pulmonary microbiota, which has not been assessed here.

In conclusion, we have identified two strains and a probiotic mix able to mitigate SARS-CoV-2 infection in hamsters. Although the mechanisms of action require further investigation, we have already shown in this study that the probiotics tested, and in particular the mix, can contribute to controlling inflammation in the lungs and improving the intestinal barrier, thereby promoting rapid recovery.

## MATERIALS AND METHODS

### *In vitro* screening of 20 bacterial strains for immunomodulatory properties

#### Experiments with PBMCs

Human PBMCs of five healthy donors (all males; aged <65 years, with a body mass index of <30; negative for HIV, hepatitis A and B viruses) were provided by Stemcell France. All PBMC experiments were conducted as described previously (19). Cytokines were quantified by ELISA after co-incubation of bacteria and PBMCs from five donors for 48 h. Strains are listed in Table S1.

#### Experiments with macrophage RAW 264.7 cell line

Murine macrophage cells 264.7 were purchased from the American Type Culture Collection. The cells were cultivated and grown in 24-well culture plates at 37°C in a 10% CO_2_-air atmosphere in Dulbecco’s modified Eagle’s medium (DMEM, Lonza) supplemented with 10% heat-inactivated fetal bovine serum (FBS, Eurobio) and 1% glutamine. The medium was changed every 2 days. Experiments started 1 day after seeding. The culture medium was changed to fresh medium with 10% heat-inactivated FBS and 1% glutamine, 0.1% streptomycin/penicillin. On the day of co-culture, bacteria were added at a multiplicity of infection of 1:40 in 50 µL of DMEM in a total volume of 500 µL. The cells were simultaneously stimulated with LPS from *Escherichia coli* O111:B4 (100 ng/mL; Sigma-Aldrich) for 24 h at 37°C in 10%/CO_2_. Samples were finally stored at −80°C until further analysis of IL-6, IL-10, and TNF-α concentration by ELISA Kit (BioLegend).

For acetate measurement, see the SCFA production section.

#### In vivo experiments

##### Experimental procedure

**Fig 6:**
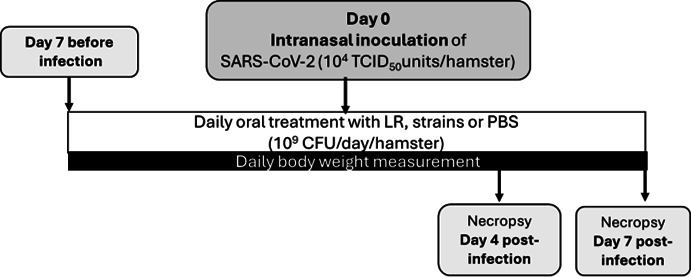
Study design. Eight-week-old female Syrian golden hamsters (strain RjHan:AURA, Janvier Breeding center, France) were housed in an animal-biosafety level 3 (A-BSL3) laboratory with *ad libitum* access to water and food. For infection experiments, hamsters were randomly assigned into five groups (*n* = 16 per group) as follows; (i) non-infected (NI) animals administered oral phosphate buffer solution (PBS, pH 7.2) (NI-PBS), (ii) infected animals (IN) administered oral PBS (IN-PBS), (iii) infected animals administered a probiotic mix (LR) (IN-LR), (iv) infected animals and oral administration of CNCM-I 5979 strain (IN-I 5979), and (v) infected animals and oral administration of CNCM-I 5644 strain (IN-I 5644). Animals were anesthetized with isoflurane and intranasally inoculated with 10^4^ TCID_50_ (median tissue culture infectious dose) units of SARS-CoV-2 strain UCN1 (GISAID reference EPI_ISL_911513) split into each nostril. Individual strains (*Bifidobacterium breve* CNCM I-5644 and CNCM I-5979, Université de Paris) and the probiotic mix (*Bifidobacterium longum* LA101, *Lactobacillus helveticus* LA102, *Lactococcus lactis* LA103, and *Streptococcus thermophilus* LA104, PiLeJe Laboratoire) were tested in a lyophilized form suspended in PBS at a concentration of 5 × 10^9^ CFU/mL prior to oral administration. Half of the hamsters per group were necropsied at 4 dpi, and the other half at 7 dpi.

Animals were weighed daily from 7 days before infection to 7 dpi (Fig. 6). Eight animals from each group were necropsied at 4 dpi, and the remaining animals (*n* = 8) at 7 dpi. For each animal, the following samples were collected: whole blood was collected in EDTA-coating tubes and centrifuged, and plasma samples were stored at -−80°C. Lungs, URT, ileum, and colon were stored at −80°C in two conditions: in DMEM (Lonza) containing penicillin and streptomycin (Lonza) at −80°C (following immediate freezing in liquid nitrogen) for protein quantification and in RNA-later (Thermo Fisher Scientific) for RT-qPCR. Fecal samples were stored at −80°C without any preservative.

##### Virus load and titration

Titration was performed on 90% confluent Vero-E6 cells in 96-well plates. Viral titers were calculated using the Spearman-Kärber method (23).

Lung RNA extraction was performed as previously described (24). TaqMan RT-qPCR was performed using QuantiTect Probe RT-PCR and E Sarbeco primers targeting the envelope protein gene (E gene). Absolute quantification was performed using a standard curve based on six 10-fold dilutions of a positive control SARS-CoV-2 RNA at 3.23 × 10^8^ copies/µL.

##### SCFA production

SCFA content was determined by gas chromatography (GC; Agilent 6890 N Network, Agilent Technologies) equipped with a split-splitless injector (GC Agilent 7890B), a flame-ionization detector and a capillary column (15 m × 0.53 mm × 0.5 μm) packed with SP 1000 (Nukol; Supelco 25236) as previously described (15). The fecal sample was extracted with water (wt g/vol), centrifuged at 12,000 × *g* for 10 min, and the supernatant collected. Fecal samples and/or acetate in bacterial supernatant samples were deproteinized overnight at 4°C by adding phosphotungstic acid (10% [vol/vol]; Sigma). A volume of 0.1 mL of the supernatant was analyzed using a gas-liquid chromatograph (Autosystem XL; Perkin Elmer). The flow rate of hydrogen, the carrier gas, was 10 mL/min; the temperature of the injector, column, and detector was 200°C, 100°C, and 240°C, respectively. 2-ethylbutyrate was used as an internal standard, and a panel of SCFA (Supelco) at 10 mM was used as the technical controls. All samples were analyzed in duplicate. Data were processed using the OpenLab Chemsation software version 2.3 (Agilent).

##### Microbiota analysis

Fecal samples were collected at 4 and 7 dpi and stored at −80°C until further analysis. Microbial DNA was extracted from 100 mg of fecal sample using the Maxwell RSC Fecal Microbiome DNA Kit on the Maxwell RSC Instrument according to the provided user guidelines (Promega). The concentration and quality of extracted DNA were assessed photometrically using a NanoDrop One/OneC UV-Vis Spectrophotometer (NanoDrop Technologies). The universal primer set 341F (5′-CCTAYGGGRBGCASCAG-3′) and 806R (5′-GGACTACNNGGGTATCTAAT-3′) were used for the amplification of the V3-V4 region of bacterial 16S rRNA gene using Illumina NovaSeq PE250 platform (Novogene).

The raw 16S rRNA sequences were analyzed using the bioinformatics pipeline FROGS (Find Rapidly OTU with Galaxy Solution) (25). After quality control depletions, affiliations were investigated using BLAST (Basic Local Alignment Search Tool) by reference to the SILVA 138 16S database. Data were filtered by retaining only sequences that were present in at least three samples and contributed 0.005% to the microbial community. Only sequences of sufficient quality (alignment of 400 bp and _0.95 coverage) were retained. The phylogenetic tree was constructed using Mafft and FastTree on the FROGS pipeline. The resulting ASV (amplicon sequence variant) table was used for subsequent statistical analysis using MicrobiomeAnalyst 2.0 (16). Samples were standardized to the same depth (16,040 sequences) before analysis. Chao1 and Shannon indexes were calculated using rarefied and normalized (cumulative sum scaling) data to characterize alpha diversity. Principal coordinate analysis of the Bray-Curtis distance followed by PERMANOVA analysis was performed to assess beta-diversity. For comparison, multiple linear regression with covariate adjustment was performed using the MaAsLin-2 package. Spearman correlations between bacterial taxa and SARS-CoV-2 infection parameters were analyzed. Correlation was considered when *P* values <0.05 after correction for false discovery rate, using the Benjamini-Hochberg procedure.

##### Fecal protease activity

Fecal protease activity was determined photometrically by using azocasein as a proteolytic substrate (19, 26). Briefly, each fecal sample (50 mg) was mixed with 1 mL of reaction buffer (0.5% wt/vol NaHCO3, pH 8.3) and homogenized. The homogenate was then centrifuged at 1,800 × *g* for 10 min at 4°C. The resulting supernatant from the fecal homogenate was incubated with 100 μL of reaction buffer and 100 μL of azocasein solution (0.5% wt/vol azocasein in reaction buffer, Sigma Aldrich) at 40°C for 20 min. The reaction was terminated by adding 100 μL of 10% vol/vol trichloroacetic acid (Sigma Aldrich). Following a second centrifugation at 1,800 × *g* for 10 min at 4°C, the absorbance of the clear supernatants was measured at 450 nm using a microplate reader.

##### RNA extraction and RT-qPCR

Total RNA was isolated, and cDNA was synthesized. The reaction mixture consisted of Takyon Low ROX SYBR 2× MasterMix blue dTTP (Eurogentec), primers at 0.5 μM, and 60 ng of cDNA. Additionally, the Syrian hamster IFN-γ TaqMan was used, mixed with 6 µL cDNA samples and TaqMan Universal Master Mix 2× (Life Technologies, USA) according to the manufacturer’s instructions. Values were expressed as normalized relative fold differences with respect to the housekeeping gene, γ-actin (TaqMan assay no. Cg04432391_mH, Applied Biosystems, USA) by the 2-ΔΔCT method. Primers are listed in Table S2.

### Statistical analyses

All results were expressed as means ± standard error of the mean. A one-way ANOVA was performed for normal samples, and multiple comparisons were carried out using Dunnett’s test. For non-normal samples, non-parametric tests were performed within groups (Kruskal-Wallis test), and multiple comparisons were carried out using Dunn’s test using GraphPad Prism 9 software (GraphPad Software). We used an alpha level of 0.05.

## SUPPLEMENTAL MATERIAL

Supplemental material aem.00648-25-s0001.docx Supplemental methods; Tables S1 and S2.

ASM does not own the copyrights to Supplemental Material that may be linked to, or accessed through, an article. The authors have granted ASM a non-exclusive, world-wide license to publish the Supplemental Material files. Please contact the corresponding author directly for reuse.
