# Supplementary material for: Marked reduction of SARS-CoV-2 infection and improved recovery following supplementation with a probiotic mix of four strains and two strains of Bifidobacterium breve in hamsters
Source: Appl Environ Microbiol. 2025 May 12;91(6):e00648-25. doi: 10.1128/aem.00648-25 (PMC12175514; doi:10.1128/aem.00648-25)
Supplement: Supplemental material — Supplemental methods; Tables S1 and S2. [file aem.00648-25-s0001.docx]

**Marked reduction of SARS-CoV-2 infection and improved recovery following supplementation with a probiotic mix of four strains and two strains of *Bifidobacterium breve* in hamsters**

**Short title: Reduced SARS-CoV-2 infection and improved recovery with probiotics in hamsters**

**Supplementary Materials and Methods**

***In vitro screening of 20 bacterial strains for immunomodulatory properties***

The list of strains is presented in **S1 Table**.

**Experiments with peripheral blood mononuclear cells** (**PBMCs**)**.** Human PBMCs of five healthy donors (all males; aged <65 years, with a body mass index of <30; negative for HIV, hepatitis A and B viruses) were provided by STEMCELL France. PBMC experiments were performed as described previously [1]. Cytokines were quantified by ELISA after co-incubation of bacteria and PBMCs from five donors for 48 h.

**Experiments with macrophage RAW 264.7 cell line.** Murine macrophage cells 264.7 were purchased from American Type Culture Collection (ATCC). The cells were cultivated and grown in 24-well culture plates in Dulbecco's modified Eagle's medium (DMEM, Lonza) supplemented with 10% heat-inactivated fetal bovine serum (FBS, Eurobio), 1% glutamine at 37°C in a 10% CO2-air atmosphere. The medium was changed every two days. Experiments were started one day after seeding. The culture medium was changed to fresh medium with 10% heat inactivated FBS and 1% glutamine, 0.1% streptomycin/penicillin. On the day of co-culture, bacteria were added at a multiplicity of infection (MOI) of 1:40 in 50 μl of DMEM in a total volume of 500 μL. Cells were simultaneously stimulated with lipopolysaccharides (LPS) from *Escherichia coli* O111:B4 (100 ng/mL; Sigma-Aldrich) for 24 h at 37°C in 10%/CO2. Samples were finally stored at -80°C until further analysis of IL-6, IL-10 and TNF-α concentration by kit ELISA (Biolegend).

For acetate measurement see SCFA production section.

***In vivo* experiments**

Animals were weighted daily from 7 dbi to 4 and 7 dpi (**Fig. 4**). Eight animals from each group were necropsied at 4 dpi and the remaining animals (n=8) at 7 dpi. For each animal, the following samples were collected: EDTA whole blood, lungs, upper respiratory tract, ileum, colon, and feces. Organs were stored frozen at -80°C (Invitrogen) in DMEM (Lonza) containing penicillin and streptomycin (Lonza), RNA-later (Thermo Fisher Scientific) or stored in 4% paraformaldehyde (Sigma-Aldrich).

**SCFA production**

SCFA content was determined by gas chromatography (GC; Agilent 6890 N Network, Agilent Technologies) equipped with a split-splitless injector (GC Agilent 7890B), a flame-ionization detector and a capillary column (15 m × 0.53 mm × 0.5 μm) packed with SP 1000 (Nukol; Supelco 25,236) as previously described [2]. The fecal sample was extracted with water (wt g/vol), centrifuged at 12,000 g for 10 min, and the supernatant collected. Fecal samples and/or acetate in bacterial supernatant samples were deproteinized overnight at 4 °C by adding phosphotungstic acid (10% (v/v); Sigma). A volume of 0.1 mL of the supernatant was analyzed using a gas-liquid chromatograph (Autosystem XL; Perkin Elmer). The flow rate of hydrogen, the carrier gas, was 10 mL/min; the temperature of the injector, column and detector was 200°C, 100°C and 240°C, respectively. 2-ethylbutyrate was used as an internal standard and a panel of SCFA (Supelco) at 10 mM was used as the technical controls. All samples were analyzed in duplicate. Data were processed using the OpenLab Chemsation software version 2,3 (Agilent).

**Microbiota analysis**

Fecal samples were collected at 4 and 7 dpi and stored at -80°C until further analysis. Microbial DNA was extracted from 100 mg of fecal sample using the Maxwell® RSC Fecal Microbiome DNA Kit on the Maxwell® RSC Instrument according to the provided user guidelines (Promega). The concentration and quality of extracted DNA were assessed photometrically using a NanoDrop One/OneC UV-Vis Spectrophotometer (NanoDrop Technologies). The universal primer set 341F (5′- CCTAYGGGRBGCASCAG -3′) and 806R (5′-GGACTACNNGGGTATCTAAT -3′) were used for the amplification of the V3-V4 region of bacterial 16S rRNA gene using illumina NovaSeq PE250 platform (Novogene).

The raw 16S rRNA sequences were analyzed using the bioinformatics pipeline FROGS (Find Rapidly OTU with Galaxy Solution) [3]. After quality control depletions, affiliations were investigated using BLAST (Basic Local Alignment Search Tool) by reference to the SILVA 138 16S database. Data were filtered by retaining only sequences that were present in at least three samples and contributed 0.005% to the microbial community. Only sequences of sufficient quality (alignment of 400 bp and _0.95 coverage) were retained. The phylogenetic tree was constructed using Mafft and Fasttree on the FROGS pipeline. The resulting ASV (amplicon sequence variant) table was used for subsequent statistical analysis using MicrobiomeAnalyst 2.0 [4]. Samples were standardized to the same depth (16,040 sequences) before analysis. Chao1 and Shannon indexes were calculated using rarefied and normalized (cumulative sum scaling) data to characterize alpha diversity. Principal coordinate analysis of the Bray Curtis distance followed by Permanova analysis were performed to assess beta-diversity. For comparison, multiple linear regression with covariate adjustment was performed using MaAsLin-2 package. Spearman correlations between bacterial taxa abs SARS-CoV-2 infection parameters were analyzed. Correlation were considered when P values <0.05 after correction for false discovery rate, using Benjamini-Hochberg procedure.

**Fecal protease activity** was determined photometrically by using azocasein as a proteolytic substrate [1, 5]. Briefly, each fecal sample (50 mg) was mixed with 1 mL of reaction buffer (0.5% W/V NaHCO3, pH 8.3) and homogenized. The homogenate was then centrifuged at 1,800 g for 10 minutes at 4°C. The resulting supernatant from the fecal homogenate was incubated with 100 μL of reaction buffer and 100 μL of azocasein solution (0.5% W/V azocasein in reaction buffer, Sigma Aldrich) at 40°C for 20 minutes. The reaction was terminated by adding 100 μL of 10% V/V trichloroacetic acid (Sigma Aldrich). Following a second centrifugation at 1,800 g for 10 minutes at 4°C, the absorbance of the clear supernatants was measured at 450 nm using a microplate reader.

**RNA extraction and RTqPCR**

Total RNA was isolated and cDNA synthetized. The reaction mixture consisted of Takyon Low ROX SYBR 2× MasterMix blue dTTP (Eurogentec), primers at 0.5 μM, and 60 ng of cDNA. Additionally, the Syrian hamster IFN-γ TaqMan was used, mixed with 6 µL cDNA samples and TaqMan Universal Master Mix 2X (Life Technologies, USA) according to manufacturer’s instructions. Values were expressed as normalized relative fold differences with respect to the housekeeping gene, γ-actin (TaqMan assay no. Cg04432391_mH, Applied Biosystems, USA) by the 2-ΔΔCTmethod. The list of primers is described in **S2 Table**.

**S1 Table**. List of strains

| **Code** | **Species** |
| --- | --- |
| PI22 | *Bifidobacterium bifidum* |
| PI41 | *Lactobacillus paragasseri* |
| PI42 | *Lactobacillus paragasseri* |
| PI43 | *Lactobacillus paragasseri* |
| PI44 | *Lactiplantibacillus plantarum* |
| PI45 | *Lacticaseibacillus casei* |
| PI46 | *Lacticaseibacillus casei* |
| PI48 | *Lacticaseibacillus rhamnosus* |
| PI49 | *Lactobacillus reuteri* |
| PI50 | *Bifidobacterium animalis* subsp. *Lactis* |
| UP1139-9 | *Bifidobacterium breve* |
| CNCM I-5646 | *Bifidobacterium longum* |
| UP1139-8 | *Bifidobacterium breve* |
| CNCM I-5979 | *Bifidobacterium breve* |
| UP1139-32 | *Bifidobacterium longum* |
| UP1139-31 | *Bifidobacterium breve* |
| CNCM I-5644 | *Bifidobacterium breve* |
| CNCM I-5645 | *Bifidobacterium longum subsp. Infantis* |
| UP1139-11 | *Bifidobacterium longum* |
| UP1139-28 | *Bifidobacterium breve* |

**S2 Table**. List of primers used in this study.

| **Primer** | **Sequence 5’ to 3’** | **Reference** |
| --- | --- | --- |
| ***γ-actin*** | Forward 5’-ACAGAGAGAAGATGACGCAGATAATG-3’  Reverse 5’-GCCTGAATGGCCACGTACA-3’ | **[6]** |
| ***Occludin*** | Forward 5’-GTGGCTTCCACACTTGCTTG-3’  Reverse 5’-GCCACTTCCTGCATAAGGGT-3’ | **[6]** |
| ***Ace 2*** | Forward 5’-TCCATTGGTCTTCTGCCATCCG-3’  Reverse 5’-AGACCATCCACCTCCACTTCTC-3’ | **[7]** |
| ***Interleukin-6; Il-6*** | Forward 5’-CCATGAGGTCTACTCGGCAAA-3’  Reverse 5’-GACCACAGTGAATGTCCACAGATC-3’ | **[6]** |
| ***Interleukin-12p40; Il-12p40*** | Forward 5’-AATGCGAGGCAGCAAATTACTC-3’  Reverse 5’-CTGCTCTTGACGTTGAACTTCAAG-3’ | **[6]** |
| ***Interferon type I; Ifn-I*** | Forward 5’-CCCACCAGATGCAAAGGATT-3’  Reverse 5’-CTTGAGCAGCCACTCTTCTATG-3’ | **[6]** |
| ***IFN- γ* (Lung)** | TaqMan qPCR assay no. Cg04455412_m1 (Thermo Fisher Scientific, USA). | Cg04455412_m1 |
| ***γ-actin* (Lung)** | TaqMan qPCR assay no. Cg04432391_mH (Thermo Fisher Scientific, USA). | Cg04432391_mH |

**Reference list**

1. Torres-Maravilla E, Holowacz S, Delannoy J, Lenoir L, Jacouton E, Gervason S et al. Serpin-positive Bifidobacterium breve CNCM I-5644 improves intestinal permeability in two models of irritable bowel syndrome. Sci Rep 2022; 12(1):19776.

2. Maillard F, Meynier M, Mondot S, Pepke F, Galbert C, Torres Maravilla E et al. From In Vitro to In Vivo: A Rational Flowchart for the Selection and Characterization of Candidate Probiotic Strains in Intestinal Disorders. Microorganisms 2023; 11(4).

3. Escudié F, Auer L, Bernard M, Mariadassou M, Cauquil L, Vidal K et al. FROGS: Find, Rapidly, OTUs with Galaxy Solution. Bioinformatics 2018; 34(8):1287–94.

4. Lu Y, Zhou G, Ewald J, Pang Z, Shiri T, Xia J. MicrobiomeAnalyst 2.0: Comprehensive statistical, functional and integrative analysis of microbiome data. Nucleic Acids Res 2023; 51(W1):W310-W318.

5. Maeda S, Ohno K, Uchida K, Igarashi H, Goto-Koshino Y, Fujino Y et al. Intestinal protease-activated receptor-2 and fecal serine protease activity are increased in canine inflammatory bowel disease and may contribute to intestinal cytokine expression. J Vet Med Sci 2014; 76(8):1119–27.

6. Sencio V, Machelart A, Robil C, Benech N, Hoffmann E, Galbert C et al. Alteration of the gut microbiota following SARS-CoV-2 infection correlates with disease severity in hamsters. Gut Microbes 2022; 14(1):2018900.

7. Ferren M, Favède V, Decimo D, Iampietro M, Lieberman NAP, Weickert J-L et al. Hamster organotypic modeling of SARS-CoV-2 lung and brainstem infection. Nat Commun 2021; 12(1):5809.
